# Clinician Job Searches in the Internet Era: Internet-Based Study

**DOI:** 10.2196/12638

**Published:** 2019-07-05

**Authors:** Shalu Gillum, Natasha Williams, Brittany Brink, Edward Ross

**Affiliations:** 1 College of Medicine University of Central Florida Orlando, FL United States

**Keywords:** personnel selection, internet, academic medical centers

## Abstract

**Background:**

Traditional methods using print media and commercial firms for clinician recruiting are often limited by cost, slow pace, and suboptimal results. An efficient and fiscally sound approach is needed for searching online to recruit clinicians.

**Objective:**

The aim of the study was to assess the Web-based methods by which clinicians might be searching for jobs in a broad range of specialties and how academic medical centers can advertise clinical job openings to prominently appear on internet searches that would yield the greatest return on investment.

**Methods:**

We used a search engine (Google) to identify 8 query terms for each of the specialties and specialists (eg, dermatology and dermatologist) to determine internet job search methodologies for 12 clinical disciplines. Searches were conducted, and the data used for analysis were the first 20 results.

**Results:**

In total, 176 searches were conducted at varying times over the course of several months, and 3520 results were recorded. The following 4 types of websites appeared in the top 10 search results across all specialties searched, accounting for 52.27% (920/1760) of the results: (1) a single no-cost job aggregator (229/1760, 13.01%); (2) 2 prominent journal-based paid digital job listing services (157/1760, 8.92% and 91/1760, 5.17%, respectively); (3) a fee-based Web-based agency (137/1760, 7.78%) offering candidate profiles; and (4) society-based paid advertisements (totaling 306/1760, 17.38%). These sites accounted for 75.45% (664/880) of results limited to the top 5 results. Repetitive short-term testing yielded similar results with minor changes in the rank order.

**Conclusions:**

On the basis of our findings, we offer a specific financially prudent internet strategy for both clinicians searching the internet for employment and employers hiring clinicians in academic medical centers.

## Introduction

### Background

The advent of the internet’s rapid widespread communication capabilities has dramatically altered job recruitment and search methodologies from the perspectives of both the potential employee and employer. As an academic medical center with many open positions across multiple disciplines, we were challenged to update our recruiting approach to reach wide audiences in an efficient and fiscally prudent manner. In a Web-based era that permits free or low-cost postings, we questioned the value of using print advertisements, postal mailings, and expensive contracted subscription or search firm recruiting services. With maturation of Web-based job recruitment and marketing, it behooves employers from multiple industries to not only invest resources in looking for prospective employees on the internet but also to develop a strategy to assure that their websites appear prominently in Web-based searches. As only a few search engines (eg, Google) are the starting point for queries by most internet users, regardless of what is being searched [1], it is important to ascertain the method of job posting most likely to yield a display on the first couple of Web pages.

### Objectives

Our College of Medicine’s recent experience was that traditional approaches of using print media and commercial recruiting firms were not only limited by budget constraints but also failed to yield optimal and rapid results. Thus, we looked at the literature seeking guidance as to the best approach for specialty and subspecialty clinician searches using the internet. In that no publications were identified that directly addressed this question, we sought to rigorously study Web-based recruitment search techniques from the viewpoint of both the employer and job seeker. We hypothesized that robust internet search engines would be able to identify job opportunities posted at no cost by employers; however, it was not clear how prominently those positions would be displayed when compared with those advertised and promoted by fee-based listing agencies. We assembled a team of faculty medical librarians, a human resource professional, and a physician to assess Web-based methods by which clinicians might be looking for jobs in a broad range of specialties and how the college should advertise clinical job openings that would yield the greatest return on investment.

## Methods

### Identifying Search Terms

To determine how clinicians could look for jobs using the internet, we selected Google for the searches based on its ranking as the most popular engine with approximately 70% of the market share [1]. To assure that we were selecting search terms that would yield results relevant to legitimate medical employment, we explored the wording and phrases that an individual might use for a query in just 2 of the clinical specialties (dermatology and endocrinology). We identified 8 terms: *jobs*, *positions*, *job openings*, *job postings*, *job advertisements*, *local Orlando jobs*, *job listings*, and *employment*. The designation for the medical field was inserted before each of these search terms, using both the name of the specialty and name of the specialist, for example, *endocrinology* or *endocrinologist*. This yielded a total of 16 searches per specialty (Table 1). As the search terms did not involve personal identifiers and the data were all in the public domain, the project is not considered human research and thus not processed by our institutional review board.

### Data Collection and Analysis

Searches were conducted, and the results were recorded from the first 2 pages of Google screens based on research that users are unlikely to view search engine results past the second page [2]. We excluded paid advertisements and image results for our analyses. Searches were conducted during weekdays at varying dates and times between the hours of 10 am and 5 pm from November 2014 to March 2015.

After the query language was finalized, the searches were then expanded to a total of 12 disciplines by including the 10 additional clinical specialties of rheumatology, podiatry, internal medicine, gastroenterology, ophthalmology, infectious disease, allergy, nephrology, neurology, and pulmonary. Searches for internal medicine and infectious disease, however, had only 8 search results each (rather than 16), as there was no appropriate *-ology* or *-ologist* suffix for those specialties (Table 1). Otherwise, the same methodology and terms were used for all these searches, and the top 20 results from each of them were recorded from the first 2 pages of results displayed by Google. We then categorized and pursued all those links to determine what the job seeker would discover at the respective websites. The links to job positions were classified as being to (1) job aggregators pulling opportunities from multiple Web sources that were of no cost to either the employer or the potential employee (eg, NoCoAg); (2) fee- or subscription-based agencies charging the employer for inclusion in their database and sometimes inviting job seekers to register their interests (eg, SubscrAg); (3) journal-based paid digital job listings (eg, SubscrJ), distinct from the option of also having them in the paper publication; and (4) society-based paid advertisements (eg, SubscrSoc). We then determined patterns to these search findings across all 12 specialties, especially as to whether paying for a listing resulted in a more prominent Web page display (eg, first screen or the top 5, 10, or 20 *hits*) compared with those from no-cost aggregators.

**Table 1:** Examples of keyword specialty search terms.

Specialty search: endocrinology	Specialty search: infectious diseases
Jobs AND endocrinology OR endocrinologist	Jobs AND infectious disease
Positions AND endocrinology OR endocrinologist	Positions AND infectious disease
Job openings AND endocrinology OR endocrinologist	Job openings AND infectious disease
Job postings AND endocrinology OR endocrinologist	Job postings AND infectious disease
Job advertisements AND endocrinology OR endocrinologist	Job advertisements AND infectious disease
Local Orlando jobs AND endocrinology OR endocrinologist	Local Orlando jobs AND infectious disease
Job listings AND endocrinology OR endocrinologist	Job listings AND infectious disease
Employment AND endocrinology OR endocrinologist	Employment AND infectious disease

### Reproducibility

There was a concern that because of the fluidity of data on the internet and unknown factors driving proprietary search engine logic, there might be considerable differences in search results over the short term. To explore this possibility, the phrase *endocrinology jobs* was randomly chosen as a test search to be repeated. These keywords were entered into Google 100 times on a Monday and another 100 times on a Tuesday during 1 week in March 2015, for a total of 200 searches. The top 20 results (ie, encompassing the first 2 search results page in Google) were recorded for every repeated query.

## Results

### Overview

In total, 176 searches were conducted for the 12 disciplines using the 8 keyword terms. The results for the first 2 pages of every search were recorded: as Google defaults to 10 results per page, this represented the top 20 for each specialty and yielded 3520 results.

There were 1760 total search results for all search terms across all specialties in the top 10 results in Google (see Multimedia Appendix 1).

### Patterns Across Search Results

Unexpectedly, our findings revealed that the following 4 websites or types of websites appeared in this first page of search results across all specialties searched: a NoCoAg, a SubscrAg, at least one SubscrJ, and a SubscrSoc website (ie, relevant to the specialty searched, such as the American Association of Clinical Endocrinologists). Of these results, a particular NoCoAg (Indeed) was identified 229 times in the top 10 (13.01% (229/1760) of the time), a SubscrAg (Practice Link) 7.78% (137/1760), 2 SubscrJ sites 8.92% (157/1760) and 5.17% (91/1760; *JAMA* Career Center and *NEJM* Career Center, respectively), and, finally, various society websites each typically in single digits but totaling 17.38% (306/1760). Combined, these websites comprise 52.27% (920/1760) of the top 10 search results for all terms searched in Google (Table 2). For-hire search firms rarely appeared in any of these queries.

The pattern was even more pronounced for the top 5 search results. There were 880 total search results in the top 5 for all search terms and across all specialties. The NoCoAg appeared 14.8% (130/880) of the time in the top 5, the SubscrAg 13.5% (119/880) the 2 SubscrJs 14.2% (125/880) and 7.6% (67/880), and the society websites totaled 25.3% (223/880). Combined, these comprise 75.4% (664/880) of the top 5 search results for all terms searched in Google (Table 3 and Multimedia Appendix 2).

**Table 2:** Top 10 search results of all specialties searched (N=1760).

Search result (type)	Search result (website)	Appearing in top 10 search results, n (%)
Society websites	Multiple, combined	306 (17.38)
No cost aggregator	Indeed	229 (13.01)
Subscription journal job e-listing	*JAMA* Career Center	157 (8.92)
Subscription aggregator	Practice Link	137 (7.78)
Subscription journal job e-listing	*NEJM* Career Center	91 (5.17)

**Table 3:** Top 5 search results of all specialties searched (N=880).

Search result (type)	Search (website) result	Appearing in top 5 search results, n (%)
Society websites	Multiple, combined	223 (25.3)
No cost aggregator	Indeed	130 (14.8)
Subscription journal job e-listing	*JAMA* Career Center	125 (14.2)
Subscription aggregator	Practice Link	119 (13.5)
Subscription journal job e-listing	*NEJM* Career Center	67 (7.6)

### Reproducibility

When the term *endocrinology jobs* was searched repeatedly, results on either a Monday or Tuesday did not vary. The same pattern as described above was evident. Google results were identical for each day’s 100 searches. The order of the search results varied slightly but not enough to break the pattern as described.

## Discussion

### Overview

Academic centers, especially the state-regulated institutions, often face recruitment challenges not encountered in the private or retail sectors. Similar to most others, our college’s human resources policies and procedures require that all faculty positions, including clinical ones, have a nationwide search and be advertised for a proscribed length of time. It is important that the process reaches a wide and diverse enough audience to meet equal opportunity guidelines. A search committee must be formed and charged with locating and recommending for hire the ideal candidate. In the past, our committees have struggled with where and how to advertise open positions; cost is of great concern, especially with many ongoing recruitments. Advertising in specialty or society journals (both print and Web) can be expensive, which is also the case with search firms.

The literature shows that the internet has a beneficial effect on job searches and, ultimately, successful employment. A 2012 study by Beard et al looked at whether internet use reduces job search costs, thus discouraging job seekers from giving up on active job searching and abandoning the labor market [3]. The study found that internet use has *significant positive effect on job search efforts* and actually reduces the chance that an unemployed individual will become disenchanted with job prospects and give up looking for a job altogether by 50%. This is likely because of the fact that one can search for jobs on the internet 7 days a week, 24 hours a day, and with only the minimal cost of internet access, if any. Job prospects are increased because geographic boundaries are minimized; one can search for employment in another city, state, or even country. Another study looking at young American job seekers found that those using the internet to find jobs had unemployment durations that were 25% shorter than those who looked for jobs solely offline [4]. Young Americans are increasingly searching the internet for employment. The Pew Research Center reported in 2011 that 24% of those aged younger than 40 years rely primarily on the internet for job information, whereas only 17% look to newspapers [5].

The internet presents unique opportunities for physician employment searches and can potentially overcome many of the logistical and financial barriers of traditional methodologies. In the medical field, where highly skilled individuals are sought for teaching and clinical positions, jobs advertisements were typically placed in printed journals, specialty and society-specific publications. Search (*headhunter*) firms were also employed, and many of those used—and still continue to use—print media to locate prospective employees.

Our historical experience as an employer was that there was a high, often prohibitive, cost associated with reaching wide audiences quickly. There was the fear that attractive potential candidates would miss time-limited advertisements in scant numbers of journals. Conversely, job seekers would risk missing our infrequent periodic or niche postings. The Web has dramatically altered that dynamic. As Frank and Taylor state, “The internet has offered ways to attack time, cost, and reach simultaneously” [6]. Employers want their positions to be found by search engines that identify low- or no-cost criteria and to be displayed on the first page or 2 of results. This is facilitated by the potential employee linking to the job description through an intermediary *aggregator*. In practical terms, it would be of the utmost importance for an employer to e-list a job in such a digital format to be picked up by a popular automated aggregator and hence then appear on the first page or 2 of search results.

Thus, our suggestion for employers with nonurgent position postings and limited budgets is to announce the job on their institution’s employment website (incurring essentially no cost). They then need to perform manual searches to check whether it was picked up by high-visibility aggregators (eg, Indeed). Suitable jobs for this approach might, for example, include primary care practitioners in large organizations with enough predicted attrition to justify always being on the look-out for new hires. If the posting is not detected by aggregators using their proprietary algorithms, then this might be accomplished by appropriately modifying the institution’s e-description or by paying the aggregator directly.

For employers with more pressing needs and larger budgets, we propose expanding searches by paying for either subscription journal listings or fee-based aggregators. Deciding between those 2 possibilities lacks clarity, especially as they have different inherent strengths, and the ultimate decider might be cost. For example, SubscrAg may also maintain a searchable database into which job seekers enter their qualifications and goals; however, employers would need the manpower to take advantage of those listings, otherwise the subscription could be a waste of money. In our case, we chose one of these subscription services (Practice Link) because we had personnel available to send (*blast*) emails to thousands of potential candidates who had expressed an interest in the specialty we were advertising. Reasons for instead choosing a SubscrJ include absence of such human resources, the market penetrance of a particular journal or its appeal to a certain type of medical position, or simply the cost. Budget allowing, we suggest advertising in both the SubscrAg and SubscrJ. The next step would be to expand the advertisements by including a fee-based specialty society posting, a SubscrSoc.

Finally, for urgent, failed, difficult (eg, limited candidate availability), or high-budget searches, serious consideration needs to be given to hiring a professional search firm. This can be much more expensive but does have its advantages: these companies typically maintain listings of attractive candidates, prescreen applicants (eg, digitally or by interview), identify an applicant pool by personally contacting leaders in the chosen field, target print mailings based on such parameters as specialty or location, and avoid the costs of large human resources departments.

The question arose during this study as to why certain sites are always in the top 10 and top 5. The answer has to do with the algorithms Google uses to determine its search results or relevancy rankings. These algorithms calculate which websites most accurately match users’ search terms [7]. For example, an individual searching for rheumatology jobs will likely find that the 5th search result will have more relevant job advertisements compared with the 19th result, which could be a low-budget portal with advertisements for mostly irrelevant jobs. Another question arose concerning why Indeed appears multiple times in the search results for a given specialty. On closer inspection, it appears that Indeed pulls job advertisements from multiple sites. According to Forbes, these *aggregator* sites, including Simply Hired, Indeed, Snagajob.com, and Beyond.com, *pull and reorganize postings from other job sites to make them easy to surf*, eliminating the need for job seekers to go to multiple corporate websites looking for job advertisements [8]. For example, our college of medicine had an open position for an endocrinologist, which it advertised on its own website, and yet the same advertisement appeared on Indeed. It is interesting to note that Monster and Career Builder, 2 sites synonymous with Web-based job advertisements, rarely appeared in the top 10 search results for any clinical specialty. These sites are clearly not the right forum for advertising or looking for clinical positions at academic institutions. It is also interesting to note that no search firms appeared in the top 5 results of any clinical specialty searched.

### Limitations of the Research

There are several known limitations to the research conducted. First, only 1 search engine, Google, was chosen to conduct the searches based solely on its popularity among the general public. A random search of 1 or 2 specialties with the chosen keywords using search engines Bing and Yahoo! did yield slightly different results as far as the order in which results appeared. This could affect the composition of the top 10 and top 5 search results. Timing is another limitation. Searches were conducted on different days and at different times over the course of several weeks. When the same search (eg, *endocrinology jobs*) was test searched on different days and times, the same overall results were obtained; however, the rank order of results varied slightly. This could affect the top 10 and top 5 search results, as it appears that Google’s search results might have substantive changes in the approximate weekly time frame, but not daily. For example, someone searching for *endocrinology jobs* today may see Indeed as the tenth search result, but someone searching with the same terms next week may see Indeed at the ninth or even eleventh search result. However, after conducting 176 searches over the course of many weeks, the top 10 and top 5 search results rarely varied. Another concern regarding timing is that job seekers who are currently employed may be limited to searching only on weekends or in the evening hours; searches were not conducted during either of those 2 periods. Finally, our search approach only identifies potential candidates who are actively looking for jobs, as opposed to search firms that can expand the applicant pool to top talent not yet in the market to change employment. Exploring differences in expertise between active and passive candidates is outside the scope of this study.

### Conclusions

#### Advice for Employers

On the basis of the results of these searches, the team conducting this study at the College of Medicine determined that the best approaches for future search committees charged with hiring clinicians at an academic institution are to (1) advertise on your institution’s own website; (2) check if an NoCoAg picked up that advertisement from your website; (3) if not, alter your advertisement accordingly or pay to advertise there; (4) consider paying for what could be costly SubscrAg services, based on whether you have the staff and resources to take advantage of contacting prospective candidates who have registered and created a career profile that contains (albeit limited) data, which could narrow down the search; (5) pay to advertise with a SubscrJ, which will depend on the reputation and competitiveness of the various journals in the particular specialty; (6) pay to advertise on 1 society website, which may be less practical when a particular field has multiple such organizations.

#### Advice for Job Seekers

On the basis of the results of these searches, job seekers should be aware of the following when searching for jobs on the internet: (1) trying multiple search engines might add a sense of completion but has diminishing returns; (2) test the search terms suggested in our tables but do not expect dramatically different results when viewing the first 2 search pages; (3) it is not likely to be productive to conduct the same search (ie, use the same keywords) multiple times per day, as you will likely get the same results—instead try again in a week’s time; (4) pay attention to patterns in search results, particularly in the top 10—sites that keep appearing likely have more relevant content; (5) it may be beneficial to create a profile in a SubscrAg, as it appears so often in the top 10 and top 5, and employers who register have access to your information.

## Appendix

Multimedia Appendix 1 Top 10 Google search results for specialist and keyword jobs.

Multimedia Appendix 2 Top 5 Google search results for specialty and keyword jobs.
