# Supplementary material for: Clinician Job Searches in the Internet Era: Internet-Based Study
Source: J Med Internet Res. 2019 Jul 5;21(7):e12638. doi: 10.2196/12638 (PMC6640069; doi:10.2196/12638)
Supplement: Multimedia Appendix 1 [file jmir_v21i7e12638_app1.pdf]

Search Terms	Result 1	Result 2	Result 3	Result 4	Result 5	Result 6	Result 7	Result 8	Result 9	Result 10
Allergist Jobs	American Academy of Allergy, Asthma, and Immunology	American Academy of Allergy, Asthma, and Immunology	Indeed	JAMA Career Center	Simply Hired	Glassdoor	Doc Café	CareerMD	All Job Openings	Practice Link
Dermatologist Jobs	Indeed	American Academy of Dermatology (from Health eCareers)	Practice Link	JAMA Career Center	Career Builder	Doc Café	LinkedIn	Monster	Career MD	Education Portal
Endocrinologist Jobs	Practice Link	NEJM Career Center	Endocrine Society	Indeed	American Association of Clinical Endocrinologists	JAMA Career Center	Doc Café	Education Portal	CareerMD	Glassdoor
Gastroenterologist Jobs	American Gastroenterological Association (from Health eCareers)	Practice Link	NEJM Career Center	Indeed	JAMA Career Center	JAMA Career Center	Merritt Hawkins	MD Search	Doc Café	American College of Gastroenterology
Nephrologist Jobs	NEJM Career Center	Indeed	Practice Link	JAMA Career Center	Glassdoor	Doc Café	Simply Hired	MD Linx	Nephrology USA	Twitter - @NephrologyJob
Neurologist Jobs	American Academy of Neurology	Indeed	Practice Link	JAMA Career Center	NEJM Career Center	Doc Café	Jackson & Coker	Career Builder	Locum Tenens	MD Search
Ophthalmologist Jobs	American Academy of Ophthalmology	Indeed	Practice Link	JAMA Career Center	Prospects	Career Builder	Locum Tenens	BMJ	Doc Café	Ophthalmology Jobs Online
Podiatrist Jobs	Indeed	Health Careers	American Podiatric Medical Association	Simply Hired	Glassdoor	Monster	American Academy of Podiatric Practice	Facebook - Get Podiatry Jobs	Podiatry Exchange	Navy
Pulmonologist Jobs	Indeed	Practice Link	ACCP Career Connection	NEJM Career Center	JAMA Career Center	Locum Tenens	Central Florida Pulmonary	MD Search	Simply Hired	CompHealth
Rheumatologist Jobs	JAMA Career Center	Indeed	Practice Link	NEJM Career Center	Amerian College of Rheumatology	Doc Café	Locum Tenens	MD Search	BMJ	Rheumatology Career

Legend

Site	Catergory
Society/Association	SubscrSoc
Indeed	NoCoAg
JAMA	SubscrJ
NEJM	SubscrJ
Practice Link	SubscrAg
