# Supplementary material for: Clinician Job Searches in the Internet Era: Internet-Based Study
Source: J Med Internet Res. 2019 Jul 5;21(7):e12638. doi: 10.2196/12638 (PMC6640069; doi:10.2196/12638)
Supplement: Multimedia Appendix 2 [file jmir_v21i7e12638_app2.pdf]

Search Terms	Result 1	Result 2	Result 3	Result 4	Result 5
**Allergy Jobs**	American Academy of Allergy, Asthma, and Immunology	American Academy of Allergy, Asthma, and Immunology	JAMA Career Center	Indeed	Indeed
**Dermatology Jobs**	American Academy of Dermatology (from Health eCareers)	JAMA Career Center	Practice Link	Indeed	Indeed
**Endocrinology Jobs**	Practice Link	Endocrine Society	NEJM Career Center	American Association of Clinical Endocrinologists	JAMA Career Center
**Gastroenterology Jobs**	American Gastroenterological Association (from Health eCareers)	Practice Link	NEJM Career Center	JAMA Career Center	Indeed
**Infectious Disease Jobs**	Health e careers	NEJM Career Center	Practice Link	JAMA Career Center	Infectious Disease Society of America
**Internal Medicine Jobs**	Practice Link	American College of Physicians	American College of Physicians	NEJM Career Center	Society of General Internal Medicine
**Nephrology Jobs**	NEJM Career Center	Practice Link	JAMA Career Center	Indeed	Indeed
**Neurology Jobs**	American Academy of Neurology	American Academy of Neurology	Practice Link	JAMA Career Center	Indeed
**Ophthalmology Jobs**	American Academy of Ophthalmology	American Academy of Ophthalmology	Practice Link	JAMA Career Center	Indeed
**Podiatry Jobs**	Health Careers	American Podiatric Medical Association	Indeed	Simply Hired	Podiatry Exchange
**Pulmonary Jobs**	ACCP Career Connection	Practice Link	Practice Link	NEJM Career Center	JAMA Careers
**Rheumatology Jobs**	Practice Link	JAMA Career Center	American College of Rheumatology	NEJM Career Center	Indeed

Legend

Site	Catergory
Society/Association	SubscrSoc
Indeed	NoCoAg
JAMA	SubscrJ
NEJM	SubscrJ
Practice Link	SubscrAg
